# Rediscovering one’s own voice in a brief psychoanalytic group intervention aimed at malignant mesothelioma patients and their families

**DOI:** 10.3389/fpsyg.2024.1471057

**Published:** 2024-12-16

**Authors:** Isabella Giulia Franzoi

**Affiliations:** Department of Psychology, University of Turin, Turin, Italy

**Keywords:** mesothelioma, cancer, brief psychotherapy, group, psychoanalysis

## Abstract

Occupational and/or environmental exposure to asbestos can lead to clinical manifestation of a variety of diseases, including malignant mesothelioma (MM), a rare cancer with a particularly high incidence rate in areas with a long history of asbestos processing. This paper aims to describe brief psychoanalytic groups (BPGs), which is an intervention model aimed at MM patients and their families in the early stages of the disease, shortly after diagnosis. The BPG model comprises 12 weekly sessions of 1 h each, co-led by two psychoanalytically oriented psychotherapists who are trained in working with cancer patients and their families and in the specifics of the BPG setting. Reflections in this paper on the BPGs will attempt to trace the voice of the group in clinical material, paying attention to its horizontal unfolding as a melodic development over time and its vertical unfolding as a harmonic interweaving between the different individual voices, which, even when opposed to each other, can find a generative interlocking of meaning. In the BPG, then, it is possible to set in motion transformations that allow one to embrace the different and diverse affective colorations of experience, evolve toward a thinking that is capable of incorporating intense emotions related to death and grief, follow healthier paths of interaction on an intrapsychic and interpersonal level, and find traces of one’s own vitality.

## 1 Introduction

It is now well known that occupational and/or environmental exposure to asbestos can lead to the clinical manifestation of a variety of diseases, such as pleural thickening and plaques, asbestosis and pulmonary fibrosis, cancers of the lung, pharynx, larynx, stomach, colon or ovary, and malignant mesothelioma (MM), a rare cancer whose incidence is particularly high in areas with a history of asbestos processing, which to date is the only proven cause of this disease (Ulla-Mari, 2023; Wilk and Krówczyńska, 2021). Despite technical and molecular biology advances, the diagnosis of MM is still complex and almost always delayed. The tumor occurs predominantly in older people, as the latency period between exposure and manifestation of the disease varies between 30 and 40 years (Carbone et al., 2019). In addition, MM has an unfavorable prognosis: the median survival time from diagnosis is particularly poor, ranging from 8 to 12 months (Huang et al., 2023; Marinaccio et al., 2018).

The impact of MM on physical health is devastating: symptoms can include pain, fatigue, sleep disturbance, loss of appetite, and even severe impairment of the respiratory function, described as “fighting for breath/gasping for air,” punctuated by increased anxiety related to the fear of suffocating or drowning due to fluid infiltrating the pleura as the disease progresses (Clayson et al., 2005; Kathiresan et al., 2010).

A diagnosis of MM also has a variety of effects on the mental health of patients and caregivers, including depression, severe anxiety and fear, denial and avoidance, social withdrawal, feelings of helplessness, guilt and shame, and anger and desire for revenge (Bonafede et al., 2020; Nagamatsu et al., 2022; Demirjian et al., 2024). Patients and family members often have to deal not only with the drama associated with the unfavorable prognosis of the disease and the lack of therapies or interventions that can lead to effective recovery, but also with the lack of adequate information about the course of the disease and the treatment process, as well as the lack of a specialized psychological care (Warby et al., 2019). In fact, the presence of clinical psychologists in oncology departments is not uniform in different countries or even within the territory of each country. Moreover, there are even fewer institutions where psychologists are specifically trained for MM patients.

For both patients and family members, this is a real-life scenario that occupies their psyche with its concrete aspects and hinders the possibility of psychological work on the meaning of what is happening. There is no space other than the mere perception of the reality of the disease to which they are helplessly exposed (Ambrosiano and Gaburri, 2013), and the future becomes a static time in which there is nothing other than the motionless waiting for the moment when the disease executes its sentence.

Lastly, in contaminated sites, i.e., areas with a history of occupational and/or environmental exposure to asbestos, the somatopsychic balance of the entire community is threatened.

The human condition is profoundly characterized by a dialectical tension between the sense of being and the expectation of non-being, often crystallized in a purely intellectual awareness of mortality that conceals a fantasy that denies its own finitude (Hoffman, 1979). Our everyday lives are often imbued with a kind of naïve realism and optimism (e.g., “see you tomorrow”). These are absolutisms based on the illusion of immortality and control over external reality that allow us to function in the world without succumbing to the anxieties that arise from the perception of our own mortality (Fraire and Rossanda, 2008; Stolorow, 1999). Living in the presence of a community phantom that sees death knocking at every door (Borgogno et al., 2015; Granieri, 2016, 2017) brings into play a knowledge of the fragility of the self that is no longer merely intellectual but, on the contrary, visceral and embodied.

Such aspects can interfere with the citizen’s ability to incorporate the affective datum into intrapsychic and interpersonal discourse, paving the way for an unconscious life characterized by the use of strong primitive defense mechanisms and thinking styles characterized by a mixture of depressive and hypomanic aspects. Indeed, people may oscillate between the contact with death anxieties, gried and loss, which can lead to depressive feelings (see, e.g., Rai and Ross, 2024), and – when this contact becomes too overwhelming – defense mechanisms such as omnipotence and denial of illness, death, and related limitations that maintain hypomanic functioning (see, e.g., McWilliams, 2011). The ability to develop thoughts rooted in reality may falter, hindering the possibility to rely on effective active strategies to cope with continuing to live in a contaminated site.

## 2 Brief psychoanalytic groups (BPGs) addressed to MM patients and their family members

The work carried out by Antonella Granieri and the Postgraduate School in Clinical Psychology of the University of Turin since 2006 in the contaminated site of Casale Monferrato has led to the definition of a psychoanalytically oriented model of clinical psychology for contaminated communities (Granieri, 2015a), which includes: (1) the joint communication of the diagnosis by the oncologist and the clinical psychologist; (2) a first level of psychotherapeutic intervention for patients and family members according to the model of BPGs (Granieri et al., 2018); (3) a second level of psychotherapeutic intervention aimed at the whole population of the community and conducted according to the multifamily psychoanalysis model (Granieri, 2016, 2017).

In the following pages, I will focus specifically on BPGs.

In planning a psychotherapeutic intervention for patients with MM and their caregivers, Antonella Granieri shared with the research-intervention group she was coordinating the idea that a brief group intervention would be the most appropriate tool to facilitate the elaboration of the impact of the disease and its physical and emotional consequences on the whole family unit. The reduced life expectancy of MM patients and the severity of their clinical condition in the last stage of the disease were decisive factors in the decision to develop a brief intervention model.

Furthermore, in a group setting psychotherapeutic interventions can be offered to several patients and family members at the same time, which limits the costs for the healthcare system. Certainly, the global economic crisis and the rising costs of the national healthcare system require a healthcare policy that takes into account the quality of the services provided and, at the same time, the need to contain costs. An important strategy to rationalize costs without compromising the quality of care is the adoption of a needs-based approach that involves the implementation of sustainable and effective interventions within a multidisciplinary approach (Schouten et al., 2016). On the one hand, the group setting makes it possible to offer a therapeutic intervention to more people at the same time, with less impact on working hours and with a reduction in waiting lists, which are also difficult to maintain given the reduced life expectancy at the time of diagnosis that characterizes MM. On the other hand, as a therapeutic tool, the group responds to the health needs of the population, being particularly effective in strengthening social ties, reducing experiences of exclusion that can occur during illness and bereavement, and restoring the family climate on which the disease can have a significant impact (Granieri et al., 2018).

In the absence of previous research suggesting the effectiveness of specific individual or group interventions for this population, the decision on how to develop the intervention was based on our theoretical frame of reference, which is psychoanalytic. In particular, we considered it important to create a setting in which the emergence of multiple narratives about somatopsychic pain is possible (Granieri, 2011), fostering the development of genuine ego resources to support the suffering parts of the self (Ezriel, 2011; Neri, 2015), and allowing patients and family members to experience new ways of being together and communicating (Granieri et al., 2018). The group is a privileged instrument for bringing about these transformations despite the short time, because while it can be difficult for a single person to grasp different perspectives on a problem, the different members of the group can bring different points of view and everyone can enjoy many perspectives at the same time. Within such a dynamic, the presence of others helps to promote the development of one’s own resources, to gain more and more courage to express oneself spontaneously and to introject new ways of functioning that were previously at work in the co-leaders and in the mind of the group (Granieri, 2017; Neri, 2021). In this intersubjective field, transference and countertransference represent communicative outposts of thoughts that are still in search of thinkers and that are taken up and transformed from time to time by a co-leader, a patient or a family member (Granieri, 2016).

For what concerns the details of the BPG model, it consists of 12 weekly sessions of 1 h each, which take place in the first months after diagnosis and are co-led by two psychoanalytically oriented psychotherapists who are trained in working with cancer patients and their families as well as in the special features of the BPG setting. The choice of 12 weekly sessions (spread over a period of 3–4 months) seemed to be a good compromise between the need to shorten the duration of the intervention, given the characteristics of MM, and the need for congruous time to allow in-depth work. The inclusion criteria for participation in the BPGs are: a diagnosis of MM with a life expectancy of at least 8 months or a family caregiver (a partner, parent, son/daughter or other relative involved in daily care) of a MM patient with a life expectancy of at least 8 months and a good motivation to participate in group therapy. The exclusion criteria are: a diagnosis of psychiatric or neurodegenerative disease, marked paranoid personality traits, other psychotherapeutic interventions, poor knowledge of the Italian language. Clinical psychologists, present at the communication of the diagnosis and at some visits to oncologists, present BPG among the treatment options and collect the interest of patients and/or caregivers. Interviews with clinical psychologists can also be requested by other healthcare professionals or by patients/carers who, at any point in their illness, feel the need for a space to cope with the impact of the disease. When at least 8 people have expressed an interest in the intervention, the preliminary interviews conducted by the therapists leading the group begin. In these interviews, the presence of the exclusion criteria is further assessed, the motivation to participate in the group is explored and the setting is explained, which is then taken up in the first session of the group. If the patient and/or family member agrees to participate in the group, he or she is contacted to complete a test battery (which is also carried out at the end of the intervention to assess the effectiveness of the group), the information sheet is handed out and the therapeutic contract and informed consent form are signed. If both the caregiver and the patient wish to participate, both must agree to participate the group together. Each BPG consists of a minimum of 10 and a maximum of 20 participants. On average, 10–15 people have participated in the BPGs so far. So far, 4 face-to-face groups and 2 online groups have taken place, and another online group is currently being set up. A total of 88 people have participated in the groups: 49 MM patients and 39 family members.

The BPG model is described in detail in Granieri et al. (2018). In brief, it comprises three phases. In the first three sessions, clinicians analyze the physical effects of the new condition (e.g., limitations, physical changes) and relate them to painful feelings that are often difficult for patients and their families to express. They explore how people deal with the sick body and its new limitations and needs, together with the unconscious desires, fears and feelings related to the diagnosis and its causes. The main aim of the first phase is to find a somatopsychic focus for the group’s work (see below). In this phase, the psychotherapists take up the participants’ contributions in order to identify and share similarities and differences between the reported experiences with the group on the one hand and to work on defining the somatopsychic focus on the other. In the second phase, the co-leaders support the group in working on the somatopsychic focus by helping members to recognize their inner states (e.g., feelings, thoughts, fantasies, desires) and relate them to everyday events, medical treatments, and emerging symptoms. The co-leaders relate explicit and implicit content back to the somatopsychic focus. They actively promote the ability to reflect on one’s own mental states and current experiences and to name the underlying defensive and dysfunctional patterns. In the final four sessions, psychotherapists help the group review their history (illnesses, absences/deaths, resources, shared strategies), explore conscious and unconscious fantasies about what the end of therapy means, and identify what they will take home from their work together, while also giving back to the group their perspective on clinical work.

At the end of each session, the psychotherapists share their perspectives on what has happened, their affective responses, the group dynamics and the dynamics of their relationship. Before each session, they take a few moments to reflect on the previous sessions and share what they feel is important for the upcoming session. There are moments of joint supervision by the two therapists, especially before establishing the somatopsychic focus and at the end of the group itself.

Above all, I would like to deepen the therapeutic work with the somatopsychic focus. This is not about tying the psychotherapy to a specific goal or specific aspects of intrapsychic and interpersonal functioning, but about using a shared image/metaphor to define a common theme that recurs throughout the sessions and relates to the disease, current symptoms, therapies and associated limitations, while underpinning specific affects and intrapsychic or interpersonal dynamics. The co-leaders facilitate the group to focus on this theme, which will then be the object of transformation in the clinical work in subsequent sessions.

We can think of the somatopsychic focus as a common attractor of meaning that is repeated across sessions and is shaped differently from session to session and from participant to participant. In the language of music, we could say that the identification of the somatopsychic focus depends on the psychotherapist’s clinical ability to capture the common theme in different variations and to bring even seemingly peripheral elements back to it. Working on the theme requires the psychotherapist to be able to follow the different melodic lines that emerge in the group, to follow the development of the different individual voices within the group over time, and to understand how the individual voices intertwine moment by moment to compose the harmony of the group. If the therapist knows how to follow both the melody and the harmony, the horizontal and vertical development of the group’s music, he/she can allow successive repetitions of the theme to be enriched with melodic lines or harmonic elements that are not yet or only partially expressed, placing the theme in a transformed and transforming sound atmosphere. Starting from the re-presentation of the theme by the different group participants, it is therefore possible to make vertex shifts that allow both a greater analytical depth and the emergence of a greater plurality of voices in the field. In this sense, the focus is characterized by being a selected fact (Bion, 1970), capable of organizing the elements in the field into a mental figure with meaning and intercepting the mental pain circulating in the group scene without seeking its relief through fragmentation and evacuation (Gaburri, 1997).

In defining the somatopsychic focus, it is important that the co-therapists can choose unsaturated linguistic solutions, imbued with sensuality, capable of stimulating affects and thoughts (Borgogno, 1997), and going beyond the phenomenology of the patient’s narrative.

Thus, in the BPG model, the somatopsychic focus emerges from the words of the participants, from the shared experiences in the group, but it is important that it is the therapists who define it and propose it to the group. Since it is a brief intervention and several themes may emerge in the first sessions of clinical work, defining the focus requires particular clinical expertise to find a metaphor that touches all participants and that the group can work with effectively in the later sessions. Inadequate definition of the focus can undermine the clinical work. If, on the other hand, the focus is well defined, participants feel that something of themselves, something from their lived experience, has been symbolized and mentalized by the psychotherapists. This allows them to feel recognized in their most personal aspects and understood in the depths of their own affective truth, which strengthens trust in the psychotherapists and the therapeutic alliance. Furthermore, it is possible for individual participants to feel that such intimate and deep aspects affect not only themselves but also other group members. This is something that captures a deeply personal yet shared aspect thanks to the continuous exchange of thoughts, affects, fantasies and body sensations (Corrao, 1995).

The dynamics of transference and countertransference must also be read through a lens that allows us to grasp the intersection of multiple levels. First of all, BPG is an intervention that takes place within a broader medical care process, and therefore what participants transfer to the co-leaders has to do both with their life history and the quality of their relationship to their own internal objects, and with the personification and actualization of what characterizes their history with caregivers. The latter partly consists in habitual qualities of the patient’s own way of experiencing the relationship, and partly acquires particular characteristics based on the nature of the relationships and narratives shared between the patient, their family members and the healthcare professionals. Such a reading vertex must be able to be coupled with a perspective that reads transference and countertransference as multiple and diluted on co-leaders and other group members, a theoretical key derived from the multifamily setting (Garcìa Badaracco, 1989, 2000). In the multifamily group, everything that is emotionally charged has something to do with transference: anyone during the session can become the object of transference for someone else, an element that—if properly considered, utilized and processed—can become an extremely important therapeutic aspect (Granieri and Borgogno, 2014). It is not possible to make every single one of these multiple transference visible, but—by working on some of them - to create a collective capacity to discover the transference dimension of each interpersonal relationship, so that each participant can think and see it first in others and then progressively in themselves (Garcìa Badaracco, 2000).

This results in a way of working that can be visualized as a kind of “extended mind” (Garcìa Badaracco, 1989), in which each person enriches the group with their own point of view: each individual contribution stimulates the group to generate associations, facilitated by the fact that those who are not directly involved in a situation can think more clearly about the relational situation that is being actualized in the group. Clinical work in BPGs also requires therapists to seek out the voice of the group, paying attention both to its horizontal unfolding as a melodic development over time and to its vertical development as a harmonic interweaving of the various individual voices that make it up and which, even if they are opposed, can find a generative interlocking of meaning. One must be able to hold together the group mind and the melodic development of the individual voices and even the individual thematic fragments within each voice, since each fragment has its own irreducible musical meaning.

## 3 Psychoanalytic work in the group

Working on the effects of illness, grief, and the recognition of trauma in its reality not only implies dealing with the catastrophic effects on patients and their families, but must also make space for the brushstrokes of residual vitality that remain in an existence marked by illness, grief, and the looming of death. This requires the co-therapists themselves to come into contact with their own way of resonating in the face of vulnerability and mortality and to rely on their own ability to cultivate an inner space that can remain alive and vital. An interplay of resonances that includes the specific unfolding of projective identifications and counter-identifications, the communication between non-removed unconscious aspects and the points of contact and distance between one’s own operational models and internal objects. This way of listening and resonating with the other also includes the possibility for the therapist to offer some brushstrokes of their own understanding of the patient at the right time, without exceeding the patient’s ability to extract something positive from what is sent back to them. The therapist should not expose the patient to music that is too far from the tonal center, but should be able to venture into unintegrated, chaotic, disorganized areas so that the patient can slowly tolerate more and more dissonance and no longer needs to evacuate it. In this way, emotional responses from the therapist can emerge, even if they are not fully metabolized, sometimes dissonant with the field, but potentially transformative toward greater thoughtfulness as they are able to include those silent areas that at first could only seem to be played out of key.

The encounter with the authentic voice of the therapist makes it possible to create a space in which each patient’s voice, with its irreducible uniqueness, can not only be there, but become itself and continue to transform, building and re-establishing over time its own tone, its own style, its own meanings, tolerating contact with the most primitive aspects of the self, with that “discordant chord that regulates the intimate as such” (Nancy, 2002). In this way, it is possible for the patient to construct a discourse that knows how to be authentically subjective, that is, that brings with it traces of the trajectories through which the individual has been able to give creative meaning to the encounter with the personal, interpersonal and transpersonal psychic happening (Corrao, 1984). This is a discourse that encompasses not only the relationship between the individual and external reality, but also the way in which the individual has learned to read and narrate this relationship with external reality and with themselves, their specific expressions in the face of sorrow and grief, but also in the face of beauty and life.

The ability to accept one’s own thoughts is comparable to the ability to be authentically free and spontaneous in musical performance, which requires a particular technical and artistic maturation. It is the study of technique, harmony, the genre you are in and the piece you are playing that allows you to improvise, to play with the music while being aware of the rules of the game and balancing between order and transgression.

### 3.1 1st movement^1^: the noise of the sick body

The visiting card of patients and family members is often characterized by a body brought to the forefront of communication and a strong affective component without, however, being able to rely on an authentic possibility of integrating the deep aspects of bodily and affective activation with the level of symbolization.

Bodily experiences and sensory processes are narrated almost exclusively through a list of symptoms. There are also several passages in which the group discusses individuals’ medical histories: how they found out they had mesothelioma, the medical protocols they were admitted to, the effects of chemotherapy, and so on. Narratives that are often inevitably lifeless, as the trauma forecloses the space for imagination and reflection and destroys the patient’s ability to participate in the process of meaning construction.

BPG 1, session 1^2^

Bruno, *patient:* Uh, yeah. I’ve already had those six chemo sessions, I’m doing a maintenance thing.

Rosangela, *patient:* Another one...I haven’t started it yet.

Bruno, *patient:* Have you already had those six yet?

Rosangela, *patient*: I have already had the six.

[…]

Clara, *patient*: He told me it’s getting smaller, I had the CT scan and now we are doing the last chemo session, next Monday.

Rosangela, *patient*: How many chemo [sessions] have you had?

Clara, *patient*: With this one, six.

Rosangela, *patient*: Then it will be the last one.

Clara, *patient*: And then I should continue with the pills.

At other times in the group work, fragments may emerge that have more to do with the emotional impact of the MM diagnosis on patients and family members. Intense anxieties often emerge, related to the difficult work of dealing with the profound changes associated with cancer pathology, both in terms of the physical effects of the disease and in terms of the relationships that each person has with their own body and with the people who relate to that diseased body.

All this happens under the phantom of death and grief that overshadows the entire landscape and paves the way for intense depressive feelings or, on the contrary, for denial mechanisms marked by mania.

BPG 1, session 1

Rino, *patient:* Five years ago, XXX passed away due to the same thing. He didn’t even get the disease treated. XXX simply never left the house again until he died.

[…]

Rosangela, *patient:* I go to sleep and say: “Who knows if I'll wake up again” And in the morning, I say: “I’m still here”

BPG 2, session 1

Matteo, *patient:* I don’t feel like a plague victim or any different from others. As I said, it was given to me, so I keep it and fight it.

BPG 3, session 1

Luca, *patient*: I did not talk about the disease, I never talk about it [...] I have to de-demonize the disease, that’s the main thing for me. [...] to forget what the illness is. [...] And the most important thing, in my opinion, is to give the illness as little weight as possible, because if you think about this illness, you get stuck in this illness, it then becomes an avalanche.

[…]

Viola, *caregiver (Luca’s wife)*: When my husband was diagnosed with mesothelioma, [...] I stood frozen, empty, I couldn’t move a hair, not a muscle, otherwise I would have exploded. I stood still, I stood there and [...] it happened that I actually exploded. Well, I started crying like a hysteric. [...] I was crying in despair and he went on and then he said, “Oh, look, I’m still alive, huh” [group laughter] And I was crying... No way. Then we drove home and I parked and I was still crying. At some point, he looked at me and said: “Whatever, go ahead, huh, I’m going to keep cheering myself up all day long.”

BPG 4, session 3

Luisella, *caregiver (Paolo’s wife)*: Before [...] you didn’t know anything, you were fine, you lived well. Then, instead, you stop making long-term plans. And you get up in the morning, you seem to open your eyes and close them, it’s day again, it’s night again.

Authentic engagement with the impact of illness in such an affective climate can be quite challenging, as the changes brought about by the diseased body can be read not so much as reality data calling for unprecedented solutions (including in the relationship), but as early signs of impending death or a kind of capitulation to the progression of pathology and the proximity of grief, or even as signs of reduced affective investment in the relationship. Furthermore, the ability to express negative affects can be hindered by a kind of cultural imperative - sometimes a familiar imperative - that forces one to think positively and often leaves patients and family members feeling ultimately alone in dealing with illness, death, and grief (Ehrenreich, 2009; Willig, 2011). This is a climate in which there is an unconscious “no-entry” (Heimann, 1975), an active prohibition of access to characters, aspects of the self, and events related to the experience, making the other person feel bad, wrong, and destructive about their experience.

BPG 1, session 1

Pietro, *caregiver (Rosangela’s son)*: That’s why then you try to tell her: try to react. Because in the meantime we’re in, you can’t beat yourself up; what we have to do we do, as the lady said, that is the treatments, the ones that are available and which right now are those, we have to do them, you can’t do any other way. You have to hope.

BPG 2, session 4

Giuseppe, *patient:* There are times in which maybe the wife tries to avoid it, and that is bad. Because you’re used to have certain reasoning, you’re used to… when you see that instead maybe she starts to agree with you and you don’t know if you’re right because maybe in another time, some time ago, you were bickering for half an hour, just to say, right? Because that’s what happens between husbands and wives, right? Now when maybe you see that… and she leaves me alone, it bothers you more than bickering.

In this scenario, thanks to the interventions of the co-therapists, seemingly off-topic but potentially transformative voices can slowly break through, expressing the need for relationships in which it is also possible to leave behind the fear and the feeling of being effectively checkmated by the illness, while at the same time opening up the possibility of processing these experiences.

BPG 1, session 2

Rosangela, *patient*: [We can talk about it] among ourselves, because we all know [...] what we have. [While] being with people who are fine and have nothing becomes difficult [...].

[…]

Claudio, *patient*: Well, talking about it I found many friends who are close to me even now, not like the friend who finds you: “ah yes, how sorry I am,” and then you don’t see him again. No.

[…]

Mirella, *caregiver (Claudio’s wife)*: There are friends who are friends and, instead, there are friends who [ignore you] and don’t even want to engage in the [discussion]. You almost take it as an offense because you say: they know and [...] they pretend they don’t know to avoid it.

[…]

Bruno, *patient*: We also have to consider that some people have already gone along these paths with their families and maybe they have been less fortunate than us, because of the shortness of time that years ago the disease had granted to some family members and because of treatments, because of the different opportunities we have now. This is the case of a very dear friend of mine who had lost his mother very early: [it was] discovered in August, by November she was gone. And once he had phoned me and said, “feet and hands forward, because I don’t know how to make this phone call here” […] and immediately he said “would you like me to phone you?” I said yes. On Wednesday he will be having dinner with me.

The shared definition of somatopsychic focus allows the body to be brought back to the fore, but opens up a narrative that does not stop at the mere reporting of the fact-trauma. A carefully defined focus brings to the stage the echoes of each person’s early relationships, as well as the ups and downs of developing the ability to tune into one’s body, but also the openness to the social, to *communitas* (Ambrosiano and Gaburri, 2013), and the possibility of rediscovering oneself as different, yet alive and active, regardless of how much time one can still share with loved ones.

BPG 1, session 3

Conductor 1: [We] had wondered if the theme to be addressed could be somewhat definable by a slogan, a kind of “I don’t dance anymore!” In the sense, precisely, just as we had seen there is someone who jumps into the dances, someone who stayed and waited, someone who stayed a little… who said to us: “but can one still dance now?” “But what if I can’t dance anymore? Will it still be good for me to dance?” [...] as if the problem could be how do you dance, for example, with an ill body. How do you make a person dance, when she is afraid - huh - right now to dance.

In this way, even the noise emanating from the sick body can be included in the group discourse by transforming it from a fact into a psychic fact endowed with expressive potential. However, such expressive power cannot be addressed on a symbolic level from the outset. At this stage of the therapeutic process, the group may still be at a proto-communicative level and does not have a stable symbolic base enabling it to express itself freely in the relationship (Gaburri, 1979). The sessions can therefore be enlivened by pre- and paraverbal forms of communication, which the therapist must intercept and transform toward a greater symbolic capacity (Flegenheimer, 1983).

### 3.2 2nd movement: recovering fragments of residual vitality

Through psychotherapeutic work in the group, it is possible for patients and family members to regain a more solid subjective position that can, on the one hand, maintain the testimony of the traumatic effects of illness and death and, on the other, bring back the possibility of feeling oneself to be a person who has the right to live and occupy an affective space (Neri, 2021). Being able to venture back into life and into the future despite illness means, on the one hand, the possibility of enduring the confrontation with the unknown, or rather with what one cannot know - death - and, on the other hand, brings into play an unprecedented palette of more or less warm, cold, intense or subdued colors with which each person discovers that they can paint the time they have left to live and spend with their loved ones. The catastrophic emotions associated with the diagnosis can circulate more freely in the group, and the moment they can be made explicit and shared, they lose much of their immediate traumatic potential. At the same time, in a climate where more authentic affective communication is possible, it is also possible to find the courage to name the aspects of the other person that are more difficult to handle or understand without fear of losing affection or closeness.

BPG 1, session 10

Conductor 1: Perhaps being able to tell yourself that you are afraid is also important.

Mirella, *caregiver (Claudio’s wife)*: Yeah, huh! I mean, you really get this tremendous heartbeat, don’t you? When I was being examined, they were saying like: “it skips a beat!”

Claudio, *patient*: That’s bizarre!

Mirella, *caregiver (Claudio’s wife)*: Then they say: “Ah! No, no, it’s just agitation, anxiety” [...] you may have been fine, however what happens to you, happens to you forever.

[…]

Clara, *patient*: Well, on the one hand that was a signal, at least one can act.

Claudio, *patient*: The signals must all be heard.

Clara, *patient*: We do not listen to them, but they are there.

Mirella, *caregiver (Claudio’s wife)*: Yes, huh! [...] And instead we act like ostriches, we put our heads under.

BPG 2, session 9

Susy, *caregiver (Ettore’s daughter)*: in spite of all the various vicissitudes of these last two years, which have been [sigh] not easy from everyone’s point of view, let’s say we are rocks and anyway between the two of us we have been growing strong with each other, with one another [...] we are on our own, on the raft like Tom Sawyer [...] every now and then there is some bad swell, we have risked a lot. Luckily, we have lost pieces, but we go on.

BPG 4, session 12

Simone, *patient*: This group has been a blessing [...] in the moments when I looked like a lion, which I’m not, and in the moments when I looked like a rabbit, which maybe I’m not even that. But guys, accept me as I am.

The group often vacillates between the feeling of being held and supported by others and the perception of their inadequacy and unreliability. At least on a conscious level, the positive pole of others’ reactions is condensed around the group members and their own affections, while the negative pole is more often related to the difficulties in dealing with the cancer treatment pathway, the experimental therapies and the events related to the recognition (and especially the non-recognition) of the disease as an occupational disease. On an unconscious level, however, it is possible that the accusation of unreliability and inadequacy is directed primarily at those who are dying, performing the extreme abandonment, or at those who find it difficult to assist a dying loved one, as well as the group that is gradually approaching its end. Indeed, the end of the psychotherapeutic journey is a reminder of impending death and grief, bringing into play a deep sense of helplessness accompanied by depressive feelings, anger and disappointment at the life that has been lived.

One area of narratives that patients often decline is the theme of unreliability in relation to their loved ones and that of the handover to those left behind (mainly their sons and daughters) in relation to certain aspects of daily life or their passions. While on the one hand there is gratitude for the care and attention received, this type of will is particularly complex because it implies recognition of impending death and unfolds in the impossibility of having sufficient time to ensure that the survivors have acquired the necessary skills to carry on the legacy of those who have gone.

*BPG 1, session 8*

Claudio, *patient*: I’ve always pruned plants, and I’m someone who, when doing one thing, several things, if I do not know, I ask first, I ask those who know. This year I did not feel like pruning, my son surprised me, he pruned them himself! When I saw that, I died [...] (laughter) But I did not say anything, I was overjoyed that he did it and stopped. The next day my uncle came and asked, “Are you afraid of the shade, here in this house?” because he saw [that he had only left] the stems [...] Now today, this plant, with a lot of effort, has started to put out flowers, a plant that used to produce “quintals” of pears will now have eight flowers... in fact, to play it down, I said to my son: “If we make a ratio, each pear will weigh four and a half kilos... for sure!”

Psychotherapeutic work within the group allows people to address these issues and regain the opportunity to bring their individual voices back into the group without excessive fear of possible dissonance, even with their own family members. This opens up the possibility of living in relationships in which love, closeness and feeling good are not created by censoring friction, but by being able to assert one’s own point of view and remaining open to encountering the point of view of others. A quality of affective regulation that makes it possible to share the fact of being alive together, to maintain contact with a sick body on a relational level, to make clear what kind of step one is able to maintain and to be able to listen to the rhythm of the other.

BPG 1, session 10

Bruno, *patient*: When I make it, I make it; when I don’t make it, I stop. [...] if you set and go to help me, then another thing takes over: that I can’t do it anymore. And I get demoralized. On the other hand, when I can’t do it, I can do it tomorrow [...] it depends on how you react to the disease: everyone has their own way of doing it, but I see it this way and it is the only way to, to react.

[…]

Claudio, *patient*: I’m going to experiment with putting on cycling shoes [...] and pointing to a toe. [...]

Rosangela, *patient*: Oh, [and you used to say] “I will never touch the bike again”

Claudio, *patient*: No, no, I put the hook in and go! I try...

An interweaving of mutual sonorities and resonances that produces a “*being-with*” (Nancy, 2002), a thought that finds the root of its own vitality precisely in the interweaving with the minds of others and with the attempts that each individual makes to make sense of the even traumatic emotions to which they are exposed without falling back into the obvious or into mass identification (Gaburri and Ambrosiano, 2003) and passing through the de-idealization of destiny as a personified, mendacious being to which the individual is necessarily inferior (Gaburri, 1997). This makes it possible to reintroduce the living into mental life and even to rejoice in its beauty and mysteriousness.

Over time, the references to somatic processes become less and less stringent, but more importantly, they can be integrated into a way of thinking that, starting from the raw affective and somatic data, knows how to incorporate these aspects into mental images and symbols, to the point where they become communicable through language (Bucci, 1994, 2007; Luborsky and Crits-Cristoph, 1990; Varvin and Rosenbaum, 2011). The body is transformed from a symptom body into a sound body, i.e., a body that is able to listen to itself and the world at the same time, one in resonance with the other. Body noises, which in the initial phase of the group are predominantly experienced as interference, can at this point be included in the spectrum of sounds that each person produces in the encounter, and as such they can be assimilated, connected. In other words, they can be transformed into narrative information for others and for oneself. In this way, they go from being noise that hinders movement to interchangeable information that allows the rhythm that makes dance possible again to be calibrated. In this way, even the most painful affective components can be better tolerated.

BPG 1, session 11

Clara, *patient*: However, I find that the fear is there. One may look in a certain way, but they have a fear! [...] When one gets a little upset, in the evening especially… I don’t know about you but [I have] crazy crises in the evening. [...] emotional crises.

Claudio, *patient*: Of thought.

Rino, *patient*: Emotional.

[…]

Rosangela, *patient*: I don’t know. Me, for example, I go to sleep... my thought is to not wake up tomorrow morning.

[…]

Claudio, *patient*: Me, more than fear, I have the certainty that I have a terrible thing inside. …

[…]

Rino, *patient*: Cry, don’t cry: it’s useless.

Rosangela, *patient*: But it is an outburst!

Rino, *patient*: I understand, but if you keep on venting your feeling like this you will get worse.

Rosangela, *patient*: But in my opinion you can’t keep it all yourself.

[…]

Rino, *patient*: [When I heard about the diagnosis, I said to myself] “Oh damn, I’m already dead” Yet here I am!

[…]

Bruno, *patient*: We all have our weaknesses, all.... What do we do, though? As I was telling him last time, some people shoot themselves, take the gun.

[…]

Clara, *patient*: You get the sorrow and anguish!

Rosangela, *patient*: It is normal. In my opinion it is normal, because one has to have [their] outburst.

[…]

Claudio, *patient*: But anguish is dangerous, huh!

Rosangela, *patient*: Huh, it’s dangerous...

Bruno, *patient*: If you fall into anguish.

Clara, *patient*: I try to cheer myself up… […] Now if I go away a little bit maybe I will have a few more distractions.

Claudio, *patient*: Settle for today, then tomorrow we’ll see.

Rosangela, *patient*: Tomorrow we’ll see!

Claudio, *patient*: Huh, good!

Clara, *patient*: We need to go for pizza.

Rosangela, *patient*: Huh, pizza will be coming.

Clara, *patient*: Pizza or whatever it is.

The farewell of and to the group offers each of the participants, as a legacy, the possibility of relying on a functioning of the psychic apparatus in which it has become possible to start thinking about one’s own emotions, affects and thoughts without necessarily falling victim to a chaos of indeterminate feelings, of *β* elements (Bion, 1962). It is a matter of setting in motion the psychoanalytic function of the mind, a function that transforms sensory impressions, emotional perturbations and mythical patterns into proto-thoughts, symbolic and linguistic images, and figures.

In this way, vivid narratives are constructed that evolve and gain meaning over time. They help to find the words to describe the effects of the illness, enrich them with meanings and connect them to all that has gone before, opening a space for a residual vitality, a “seed of the future that is germinated and generously maintained by giving it credit” (Borgogno, 1997, my translation).

BPG 4, session 12

Giorgio, *patient*: [The] experience was strong […].

Elena, *caregiver (Giorgio’s wife)*: You’re not alone.

[…]

Lorenzo, *patient*: […] Ah, you are finished if you stand there alone.

Elena, *caregiver (Giorgio’s wife)*: You look lonely, but instead...

Giorgio, *patient*: So, I know your problems, you know mine.

[…]

Lorenzo, *patient*: Yes, we talk about the same thing, we understand each other.

Giorgio, *patient*: […] We’ve talked a lot.

Simone, *patient*: Huh, it’s beautiful, really beautiful.

### 3.3 3rd movement: the re-vitalizing function of the therapists

Referring to a patient with an unfavorable prognosis that left him with a very short time to live, Bion (1977) reflects on how some patients are said to be dying, when in fact this is true of all of us humans, since we are in fact living. More important is the question of whether the life and space left to live are such that the patient feels they are worth living and in what way they can fan the flame of their residual vitality so that they can live the life they have left to live.

The perspective of death dismays on the one hand, but at the same time activates the psychoanalytic function of the mind, as it leads to the edges of limitation and transformation and brings into play the need for constant reflection on the helping function that psychotherapeutic work can offer and that allows the patient to give meaning to life despite their approaching death or perhaps precisely as a function of it (Ambrosiano, 2022). In such situations, the therapist’s task is to restore even small spaces in the patient’s mind where he or she can once again harbor that urge to exist that allows even the most painful experiences to be processed and paves the way for the feeling that life has meaning despite everything. For this to be possible, the patient must be able to recognize in the therapist another who is able to bear their anguish, to trust in existence without denying or avoiding its dark side, in which the human being is lost (Ambrosiano and Gaburri, 2013).

Faced with psychic death and the prospect of the death of the body, the psychotherapist must be able to inhabit, embody and process the realm of non-existence without trying to alleviate their own mental suffering or that of their patients by introducing artificial elements of life (Borgogno, 2011).

It is not about explaining the traumatic reality that the patient has experienced, nor is it about the analyst simply showing that they have shared the patient’s narrative. Rather, it is about the analyst having the opportunity to experience within themselves recognizable emotions that make sense of the experienced reality, rather than the generalizing, intrusive and despotic confusion experienced by the patient (Granieri, 2003).

For this to be possible, to get in touch with the frozen affects brought into the field by the diagnosis, one must train one’s “third ear” (Reik, 1948) to grasp the verbal and nonverbal nuances of the patient’s communication and the underlying tones associated with them, and to recognize the same elements in one’s own idiosyncratic response to the patient’s communication. In this way, it is possible to revitalize the contact between the outer and inner worlds and offer images and words that can transform reality into a psychological reality.

And not only that: as Borgogno (1999) points out, the patient also needs a therapist who values their attempts to come to terms with their experience of the illness and at the same time reminds them: “I am still here. I have listened to you. I understand what you are talking about. You are not the only person who has felt this way. You are not incomprehensible. I am not shocked. I’m not admonishing you or trying to get you to conform to my ideas about how you should feel or behave” (Rycroft, 1956).

With this implicit communication, the psychotherapist shows their interest and their ability to understand the patient by giving them the possibility to be themselves and to have a relationship with another person without violating their personality and their development possibilities. It is a work that allows an emotional punctuation of what happens in the group and in the participants’ lives outside the group: “a kind of vision that can incorporate emotions and affects from the phenomenology of trauma” giving “eyes, voice and human gestures to illness and death” (Neri, 2016, my translation). Therein lies the opportunity for the co-therapists to be revitalizing in relation to the group, to foster the ability to move forward despite the trauma, to contain previously unknown and unthinkable aspects of the experience, and to open the space for a fuller understanding of one’s life and personality. Knowing that, as Margaret Little points out, it has not been possible to do otherwise (Little, 2002).

In order for such transformations to be initiated, psychotherapists must work on their own countertransference, which can be particularly intense when dealing with illness and death. The sense of helplessness and the amount of psychic pain one is confronted with can lead to defense mechanisms and automatisms even in those who strive for an attitude of authentic listening, to moments in which affective involvement is rather superficial and communication takes place on a level that we could call pedagogical and prescriptive, putting the search for authentic contact in parenthesis (Borgogno, 1999). It can be difficult for psychotherapists to find a balance within themselves that allows them to contain intense, overwhelming affects without silencing them on the one hand and amplifying them on the other. Moreover, since the transference dynamics in the group have to do with both the significant relationships in the participants’ life histories and the dynamics that are brought into play with the health professionals involved in their treatment, it can be complex to manage the triangulations that arise. Indeed, sometimes aspects concerning the relationship with other healthcare professionals are projected onto the psychotherapists, sometimes they find themselves personifying the anger and negative feelings that arise in the field in relation to other professionals, sometimes they find themselves identified with other healthcare professionals when addressing patients and family members, and sometimes they have to deal with the dynamics of idealization and devaluation that they can face either specifically or as part of the healthcare team. Particularly intense are the moments when group members do not show up for the session, either because they have decided to no longer participate in the group (a rare case, but it has happened), as both psychotherapists and patients can feel betrayed by those who have decided to leave the group, or when this is due to a deterioration in health, as the specter of death comes to the fore. Very sensitive moments are also the death of a participant during a group and the communication of a diagnosis to a caregiver. These moments imply a deep contact with feelings of helplessness, uncontrollability (although the inclusion criteria take into account an estimate of survival time, the disease, like life itself, is not completely controllable) and the transience of life, not only for those affected by MM, but for all human beings. Faced with a pathology with such an infamous prognosis, psychotherapists need to work on countertransferential dynamics that can lead them, on the one hand, to pretend that they are not in the presence of a disease that leads to death and, on the other hand, to be able to say exclusively that death is the only prospect and that there is nothing more to say than that MM patients will die shortly afterwards, that caregivers will lose them and that eventually we will all die. The psychotherapists’ elaborative work enables them to move out of this psychic impasse and into a position where death is certainly in the background, where they can bear witness to the fact that although the other person is dying, they are still alive until then and are not just a body with its own physiology, but a somatopsychic unit that senses, feels, gives meaning, bonds, thinks, communicates and dreams.

## 4 Discussion

For numerous patients, young and old, it turns out that psychotherapeutic work aimed at instilling the belief in their potential for recovery proves to be paramount (Vallino, 2005). However, this goal seems paradoxical and unattainable when dealing with patients whose real-life circumstances involve death and grief.

The transformative potential of clinical intervention with cancer patients and their family members lies primarily in the possibility of moving beyond the diagnosis, the disease, transcending the frozen, incandescent traumatic affects, in search of the remaining fertile psychic soil in which the seeds of residual vitality can germinate.

From a therapeutic point of view, one often wavers between the need to free the patient from the narcissistic closure resulting from the body’s illness and the possibility of supporting their will to live toward a future that is still possible, even if it is limited by the final termination of a death that seems closer than one can bear. The possibility of becoming passionate about one’s existence implies a path that is by no means easy or painless, especially when the life one is passionate about comes to an end or opens up to a painful loss (Granieri, 2015b): it is about taking on the natural drives of life without being overly burdened by the noise of the sick body, working through the fear of existing as separate individuals and, at the same time, the fear of being absorbed into the impersonal, of losing one’s own body and therefore one’s own thoughts. It is a matter of pausing for a necessary and sufficient time and waiting for the therapeutic encounter to open up to the possibility of an authentic new beginning (Balint, 1968). This is a transformation that, in the case of a brief intervention, can only be focused but is no less vital. Such a therapeutic approach makes it possible to move from sharing fragments of sensoriality toward configurations capable of attributing meaning to one’s own disease state, but also to the relational environment and to some aspects of somatopsychic functioning (Granieri, 2016). Such a transition cannot rely solely on the patient’s sensory perceptions, which are also foregrounded, but requires a mind capable of creating images from them and mentalizing them in the dialectical play of narratives constructed in the session. It is the ability to develop a sense of tenderness (Gaburri, 2011) toward oneself and others that makes it easier to go on living and less traumatic to have to separate from loved ones, from ourselves as we were and as we will not become again (Neri, 2015).

The psychotherapeutic group can therefore offer itself as a place where listening to the specific personal modalities through which despair and powerlessness related to the catastrophic effects of the diagnosis are expressed, offers the possibility of sharing a closeness that can restore trust in oneself, in others and in life (Ambrosiano, 2022). In the words of Tonia Cancrini (2002, my translation): “In this rough and arduous human journey, however, I believe that the most important thing is to find in ourselves, however things evolve, the energy and vitality that allow us to face existence in the best and most creative way.”

## Data Availability

The raw data supporting the conclusions of this article will be made available by the authors, without undue reservation.

## Footnotes

^1^The commentary on the clinical material is divided into sections called “movements,” a term borrowed from musical language.

^2^The words of the MM patients and their caregivers are the English translation of the verbatim transcripts of the group sessions of the four face-to-face BPGs I conducted from 2015 to 2018. I have chosen not to use the material from the sessions of the groups that were conducted online, as this aspect of the setting would require further investigation, which will be deferred to future publications. The groups were conducted in rooms provided by the hospital where the patients were in charge. For all interventions, informed consent was obtained from participants for audio recording of the sessions. The sessions were audio and video recorded with a GoPro camera. The names of the individuals are fictitious, both for ethical reasons and out of respect for patient privacy, as required by the EU Regulation 2016/679 (General Data Protection Regulation - “GDPR”) as amended and supplemented.
